# Assessment of biomimetic materials in strengthening root canal-treated teeth during orthodontic intrusion or extrusion

**DOI:** 10.6026/973206300214153

**Published:** 2025-11-15

**Authors:** Suruchi Sisodia, Raksha Jain, Osama Magdy Monir Mostafa, Neeti Mittal, Priyatam Karade, Anil kumar

**Affiliations:** 1 Department of Conservative Dentistry and Endodontics, Modern Dental College and Research Centre, Indore, Madhya Pradesh, India; 2 Department of Endodontist, Almoosa Specialist Hospital, Alahsa Kingdom of Saudi Arabia; 3 Department of Pediatric and Preventive Dentistry, Institute of Dental Studies and Technologies, Modinagar, Ghaziabad, Uttar Pradesh, India; 4 Department of conservative dentistry and endodontics, Bharati Vidyapeeth, (Deemed To Be University) Dental College and Hospital, Sangli, Maharashtra, India; 5 Department of conservative dentistry and endodontics, Vasantdada patil dental college Kavalapur sangli Maharashtra-416306, India

**Keywords:** Biomimetic materials, root canal-treated teeth, fracture resistance, orthodontic intrusion and extrusion, fiber-reinforced composite

## Abstract

The biomechanical function of teeth with endodontic treatment is directly impacted by the restorative material chosen. It has been demonstrated that biomimetic restorative techniques, such as fiber-reinforced composites, polyethylene fibre reinforcement, and bioactive core materials, more successfully mimic the functional characteristics of natural dentin and enamel than traditional composites. Therefore, it is of interest to evaluate the strengthening effect of biomimetic restorative materials on root canal-treated teeth subjected to orthodontic intrusion and extrusion. Forty extracted maxillary premolars were restored using short fibre-reinforced composite, polyethylene fibre with composite, biodentine, or conventional composite, and tested under 150 g orthodontic force. Teeth restored with fibre-based materials exhibited significantly higher fracture resistance than biodentine and control groups (p < 0.05). Favorable fracture patterns predominated in fibre-reinforced groups, while conventional composites showed mainly unfavorable fractures. Biomimetic restorations, particularly fibre-based materials, enhance fracture resistance and promote favorable failure modes during orthodontic loading.

## Background:

Managing teeth that have received endodontic treatment and are undergoing orthodontic movement poses a special clinical challenge, especially when those teeth are being subjected to intricate forces like extrusion or intrusion. Root canal-treated teeth frequently show altered structural integrity because of dentin loss, changes in moisture content, and decreased proprioceptive feedback, in contrast to vital teeth, which usually show predictable biomechanical responses. Since their long-term prognosis and capacity to tolerate orthodontic forces may differ greatly from those of their vital counterparts, updated guidelines stress the importance of carefully evaluating traumatised and endodontically treated teeth clinically and radiographically before beginning orthodontic therapy [1]. Therefore, in treatment planning, interdisciplinary decision-making between endodontists and orthodontists is essential. This kind of cooperation guarantees that orthodontic biomechanics, periodontal support, and structural reinforcement are all meticulously combined to maintain the tooth's longevity and function. Because restorative materials are essential for reducing stress concentrations during orthodontic loading, clinical protocols should take into account both the tooth's endodontic condition and the restorative reinforcement strategy used [2]. The stability of teeth that were previously treated with traditional root canal therapy or regenerative endodontic procedures can be greatly impacted by orthodontic forces, according to new clinical data. In order to avoid catastrophic failures like vertical root fractures or sub-crestal crown fractures, it is crucial to use reinforcement techniques that preserve both root and coronal stability during active orthodontic movement [3]. The biomechanical function of teeth with endodontic treatment is directly impacted by the restorative material chosen. It has been demonstrated that biomimetic restorative techniques, such as fiber-reinforced composites, polyethylene fibre reinforcement, and bioactive core materials, more successfully mimic the functional characteristics of natural dentin and enamel than traditional composites. These materials help to improve fracture resistance under orthodontic stress and functional loading by improving stress distribution [4]. Short fiber-reinforced composites are particularly important among them. It has been demonstrated that their three-dimensional fibre network outperforms traditional composites by lowering stress concentration and slowing the spread of cracks, giving structurally compromised teeth better reinforcement. For endodontically treated teeth that are exposed to orthodontic intrusion or extrusion forces, where structural resilience is crucial, this makes them extremely beneficial [5]. Therefore, it is of interest to describe the effect of different biomimetic restorative materials on the fracture resistance of endodontically treated teeth under orthodontic forces.

## Methodology:

The goal of the current static methodological study was to assess how biomimetic restorative materials affected the strength of teeth that had received root canal therapy when exposed to orthodontic intrusion and extrusion forces. For this purpose, 40 maxillary premolars that had recently been extracted and were recommended for extraction for orthodontic reasons were gathered. To make sure there were no cracks, cavities, or structural flaws, every specimen was examined under a microscope. To reduce variability, teeth with comparable crown and root dimensions were included. The chosen specimens were divided into four experimental groups, each consisting of ten teeth, at random. All 40 teeth received standardised endodontic treatment using AH Plus sealer, gutta-percha, lateral condensation obturation, and rotating instrumentation. Four distinct biomimetic techniques were used to complete post-endodontic coronal restorations, and access cavities were prepared in accordance with standard protocols. The restorative material in Group I was short fibre reinforced composite (SFC), which was applied gradually in accordance with the manufacturer's instructions. In Group II, polyethylene fibres were added to the composite resin to improve fracture resistance by simulating the reinforcing action of natural dentin collagen. Because of its bioactivity and dentin-like mechanical characteristics, biodentine was utilised as a core build-up material in Group III. Lastly, Group IV was the control group, in which no extra biomimetic reinforcement was used and a traditional composite resin restoration was used. To guarantee stable positioning during testing, all specimens were embedded in acrylic resin blocks after restoration. To create a more clinically relevant model, a thin layer of silicone-based impression material was applied around the root surfaces to mimic the viscoelastic characteristics of the periodontal ligament. A universal testing machine was used to simulate orthodontic forces by applying a constant static load of 150 g in both intrusion and extrusion directions. In order to simulate the clinical situation of orthodontic tooth movement in teeth that had received root canal therapy, these forces were maintained for a predetermined amount of time. The specimens underwent compressive fracture testing at a crosshead speed of 1 mm/min until failure following load simulation. The findings showed that the four groups differed significantly from one another. The highest mean fracture resistance was shown by teeth restored with short fibre reinforced composite (Group I), which was closely followed by teeth reinforced with polyethylene fibres (Group II). When compared to both Biodentine (Group III) and the traditional composite resin group (Group IV), these two groups demonstrated statistically significant superiority. Even though fiber-based techniques were more successful, biodentine still showed better resistance than traditional composites, indicating that its biomimetic qualities help strengthen weak tooth structure. When it came to reinforcing endodontically treated teeth under orthodontic forces, the control group, which was restored using conventional composite resin, showed the lowest mean fracture resistance. To ascertain whether fractures happened in a favourable (repairable) or unfavourable (non-repairable) way, failure mode analysis was carried out in addition to fracture resistance. Unfavourable fractures extended below the cemento-enamel junction and frequently involved root-level fractures that harmed the tooth's prognosis, whereas favourable fractures were defined as those that occurred above the CEJ and are more amenable to retreatment or restoration. Favourable fractures were more common in Groups I and II (fiber-based biomimetic strategies), which is indicative of their improved capacity to disperse stress throughout the restored structure. Group IV (conventional composite) had the highest percentage of catastrophic, irreparable failures, whereas Group III (Biodentine) displayed a mixture of favourable and unfavourable fractures. When combined, the results of this static methodological study provide compelling evidence for the use of biomimetic restorative techniques to strengthen teeth with root canal therapy that are exposed to orthodontic intrusion and extrusion forces. The highest increase in fracture resistance and more advantageous failure patterns were obtained by the addition of fibres, either as polyethylene fibre reinforcement or short fibre reinforced composite. Although less successful than fiber-based methods, biodentine still performed better than traditional composites and is a good choice for biomimicry. The study concludes that choosing restorative materials with biomimetic qualities is essential for maintaining the structural integrity of teeth that have had endodontic treatment during orthodontic treatment, when additional functional stresses may be experienced.

## Results:

After undergoing standardised orthodontic load simulation and fracture resistance testing, all 40 specimens were successfully restored in accordance with the designated restorative protocols. During the experimental procedures, no specimens were lost or excluded. Table 1 (see PDF) displays the average fracture resistance values for each of the four experimental groups along with their standard deviations. With a mean value of 1215.4 ± 85.6 N, teeth restored with short fiber-reinforced composite (Group I) demonstrated the highest fracture resistance among the tested groups, demonstrating their superior reinforcing ability. Although Group II's polyethylene fiber-reinforced composite resin had somewhat lower values (1138.2 ± 92.4 N), the results were still statistically similar to Group I's (p > 0.05). According to these results, the structural resilience of teeth that had endodontic treatment was considerably increased by both fiber-based biomimetic restorative techniques. On the other hand, the mean fracture resistance of teeth restored with Biodentine as the core material (Group III) was 987.6 ± 74.8 N, which was higher than the control group but much lower than Groups I and II. With a mean value of 842.1 ± 69.3 N, the control group (Group IV) that was restored using conventional composite resin had the lowest resistance. Significant differences between Groups III and IV and Groups I and II were confirmed by statistical analysis (p < 0.05), and Table 1 (see PDF) shows these differences in clear superscript letters. Table 2 (see PDF) summarises the distribution of failure modes. With 80% and 70% of specimens exhibiting repairable fracture patterns, respectively, Groups I and II showed a preponderance of favourable fractures (above the cemento-enamel junction [CEJ]). A mixed response to loading was indicated by Group III's equal distribution of favourable (50%) and unfavourable (50%) fractures. On the other hand, Group IV had the greatest percentage of unfavourable fractures (70%)-the majority of which extended below the CEJ and were categorised as catastrophic. All things considered, the findings unequivocally demonstrate that biomimetic restorative techniques involving short fibres or polyethylene fibres enhanced the fracture resistance of teeth that had received root canal therapy and changed the failure mode to more advantageous patterns. This implies that when teeth are exposed to orthodontic loading forces, fiber-reinforced biomimetic materials offer better protection against catastrophic failures than either biodentine or traditional composite resin.

## Discussion:

In order to ensure the long-term survival of endodontically treated teeth, especially when they are exposed to functional or orthodontic stresses, reinforcement is still essential. By simulating the biomechanical characteristics of natural dentin, short fiber-reinforced composites have been extensively investigated for their potential to increase fracture resistance. It has been demonstrated that their combination with polyethylene fibres greatly increases fracture resistance, particularly in teeth with compromised structural integrity [6]. Similarly, in vitro studies have shown that direct or indirect short fiber-reinforced overlays exhibit favourable fracture behaviour in comparison to traditional composite restorations, indicating their potential as biomimetic substitutes for fortifying teeth that have undergone root canal therapy [7]. In addition to composites, polyethylene fibres have been shown to transform restorative dentistry by offering biomimetic dentin reinforcement, which functions as an internal splint to withstand both, functional and parafunctional stresses [8]. The ability of bioceramic materials, like Biodentine, to restore endodontically treated teeth has been assessed; they exhibit fracture resistance that is on par with or better than that of resin-modified glass ionomer and composite core build-ups. According to these results, biomaterials based on calcium silicate may be used as intraradicular reinforcements with good results [9]. Furthermore, it has been determined that placing intraorifice barriers is another way to increase fracture resistance, with some materials performing better than others under simulated loading conditions [10]. Biomimetic reinforcement techniques are crucial for structurally weaker roots, as studies on immature roots have shown that using Biodentine in combination with fibre posts can improve fracture resistance as ageing occurs [11]. Comparing endocrowns to traditional crowns, systematic reviews have shown that they are more effective at maintaining tooth structure and improving fracture resistance, making them a viable restorative option [12]. The choice of restoration has been closely associated with the survival of teeth that have undergone posterior endodontic treatment. Although full-coverage crowns have higher fracture resistance and survival rates than composite restorations, systematic reviews show that resin-based biomimetic techniques still have potential in conservative situations [13]. Key factors influencing the longevity of restorations have also been found to be preparation design and restorative thickness; minimally invasive preparations with optimal ceramic or composite thickness can greatly increase fracture resistance [14]. The structural integrity of endodontically treated teeth can be strengthened by experimental etching agents that can increase adhesive bond strength and encourage dentin remineralisation, thanks to recent developments in biomimetic mineralisation techniques (Yao *et al.* 2024) [15]. These methods fit in with the larger movement to incorporate biomimetic concepts into restorative dentistry, in which materials are created to mimic the morphological, functional, and aesthetic properties of natural tissues [16]. To further increase fracture resistance, fibre insertion techniques have also been investigated. It has been demonstrated that adding glass fibres to composite restorations greatly strengthens premolars that have undergone endodontic treatment; the location of the fibres affects the final mechanical result [17]. All of these results highlight the importance of biomimetic restorative techniques in strengthening endodontically treated teeth, from fibre posts and bioceramics to polyethylene fibres and short fiber-reinforced composites. In orthodontic intrusion or extrusion, where teeth are exposed to continuous mechanical stresses that could shorten their lifespan, this kind of reinforcement is especially important. Kumar *et al.* [18] highlighted that biomimetic materials mimic the mechanical behavior of natural dentin. They enhance stress distribution during functional or orthodontic loading. This reduces fracture risk and improves structural integrity in compromised teeth. Katanaki *et al.* [19] showed that clear aligner therapy can affect root length in treated teeth. Loss of dentin and altered tooth biomechanics increases susceptibility to resorption. Reinforcement with biomimetic materials is crucial for maintaining stability.

## Conclusion:

Under orthodontic forces, teeth with root canal therapy are stronger and more resilient thanks to biomimetic restorative techniques. Superior reinforcement and advantageous failure modes were demonstrated by fiber-reinforced composites and polyethylene fibres. These substances offer a viable means of improving the prognosis during orthodontic extrusion and intrusion.
